# Supplementary material for: Influence of Manufacturing Process on the Microstructure, Stability, and Sensorial Properties of a Topical Ointment Formulation
Source: Pharmaceutics. 2023 Aug 28;15(9):2219. doi: 10.3390/pharmaceutics15092219 (PMC10536044; doi:10.3390/pharmaceutics15092219)
Supplement: Supplementary file 1 [file pharmaceutics-15-02219-s001.zip › pharmaceutics-2560194-supplementary.pdf]

Supplementary Information

Influence of Manufacturing Process on the Microstructure, Stability, and Sensorial Properties of a Topical Ointment Formulation

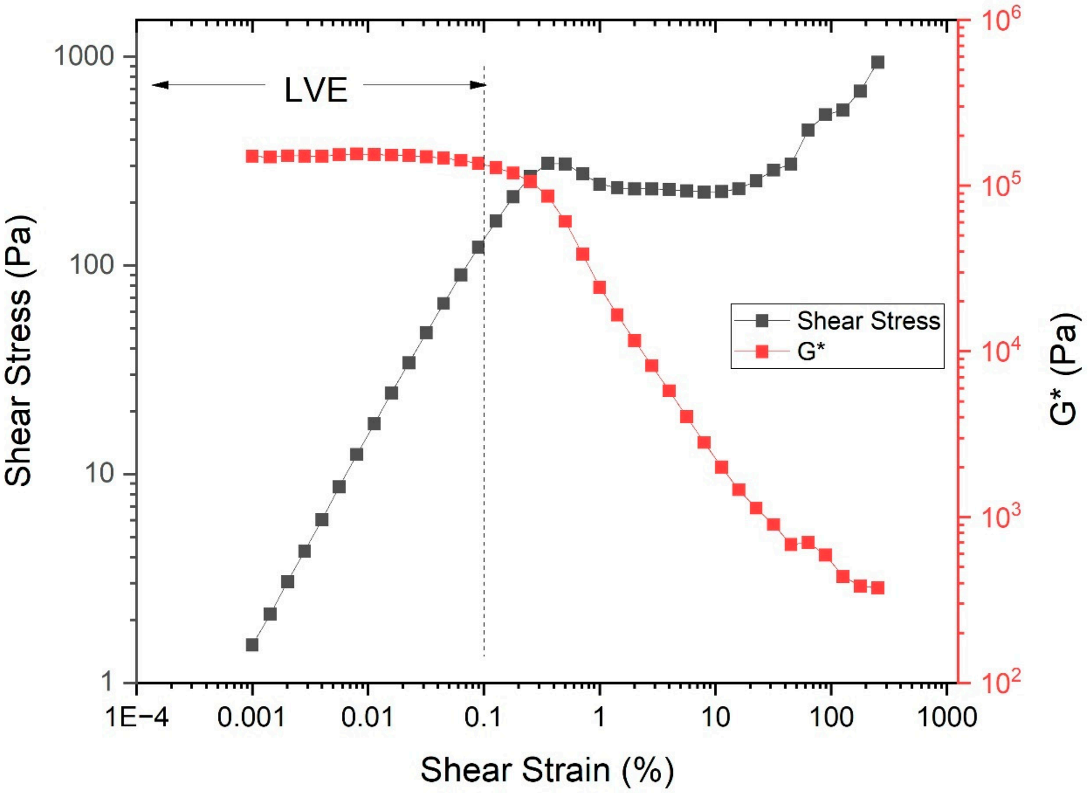

Figure S1. Complex shear modulus (G*) and shear stress as a function of shear strain for a typical ointment sample.

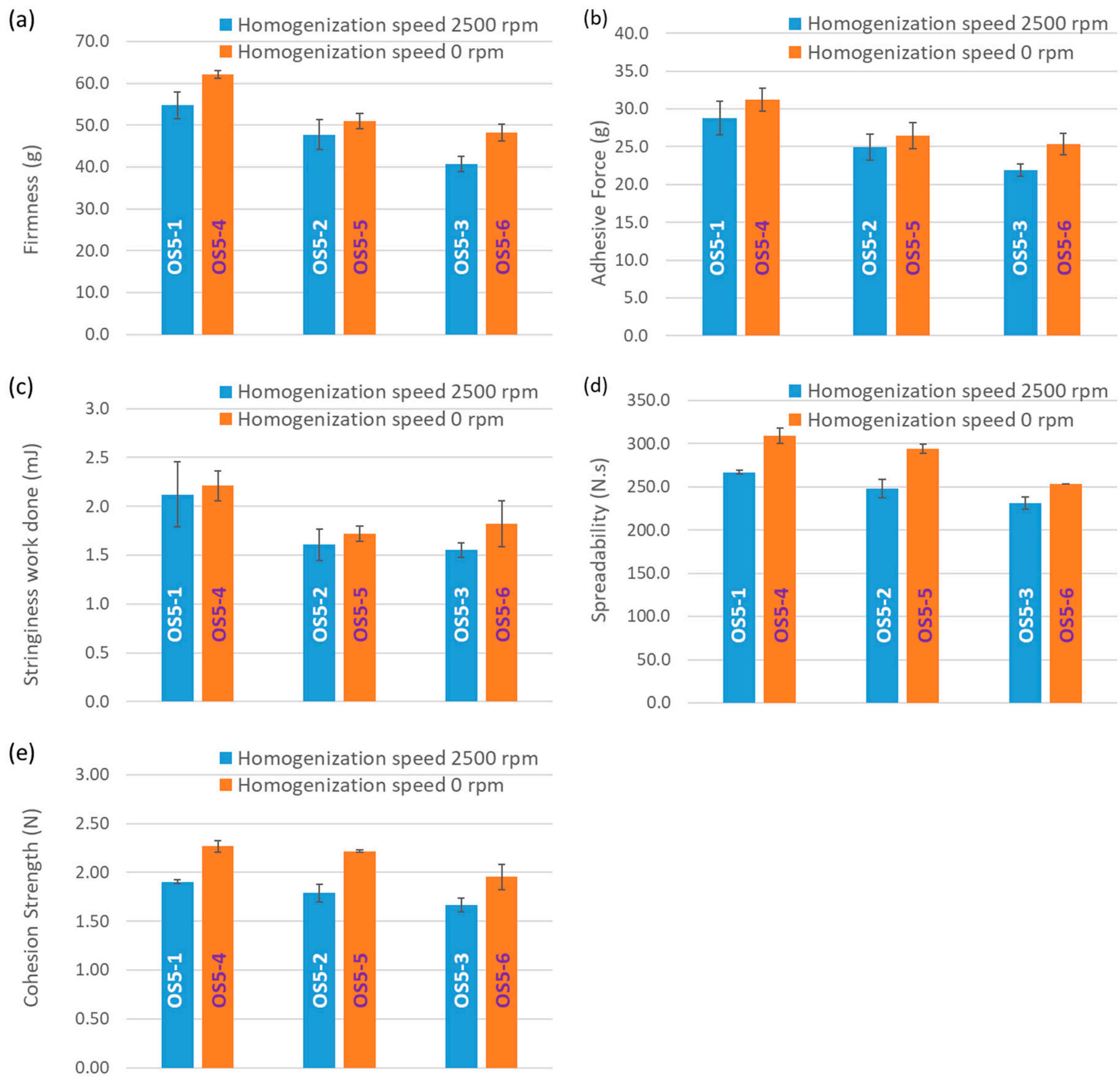

Figure S2. (a–e) Effects of step 5 homogenization speed on the textural properties of ointments.

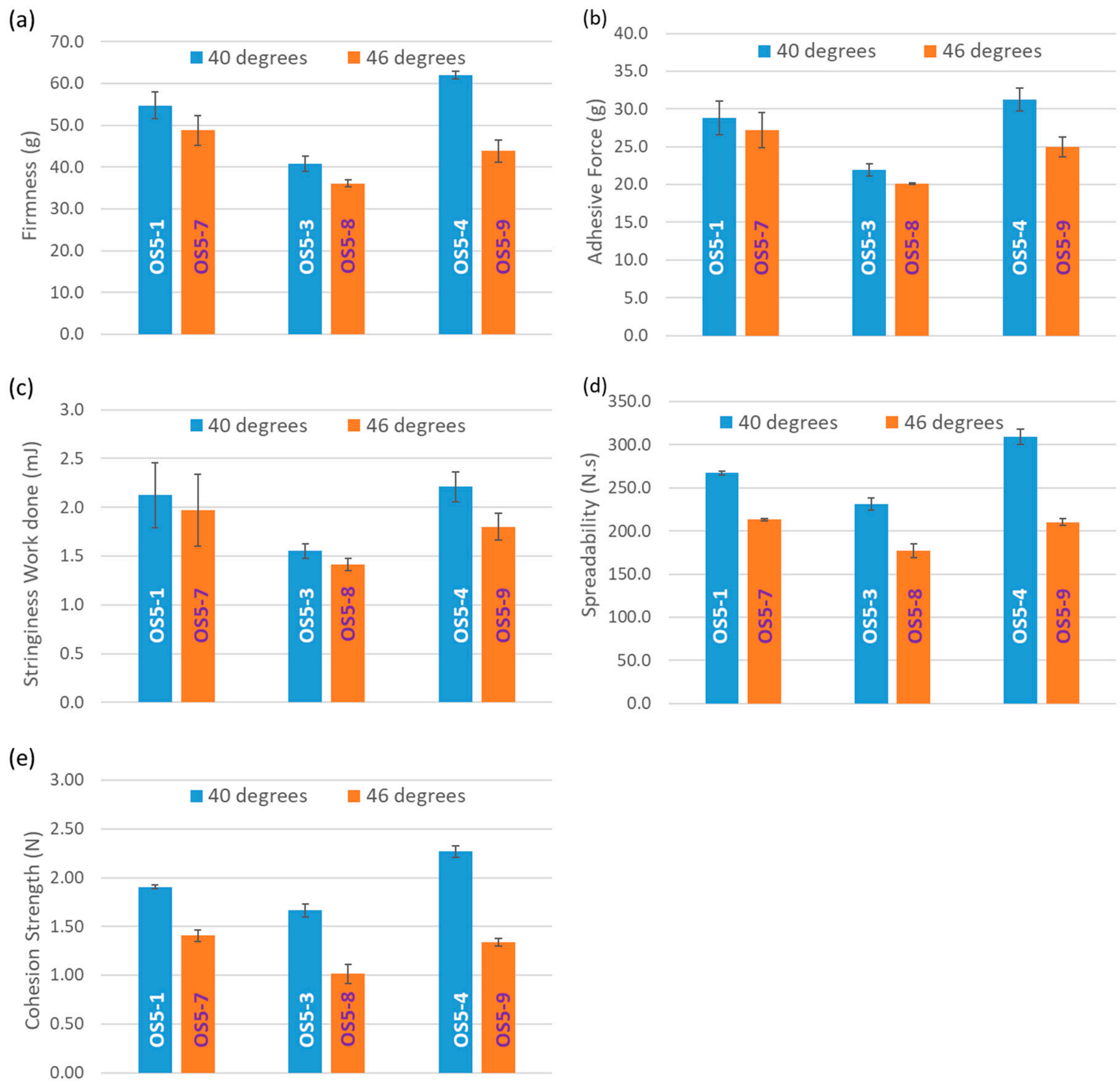

Figure S3. (a–e) Effect of PG addition temperature ([F3]) on textural properties of the ointments.

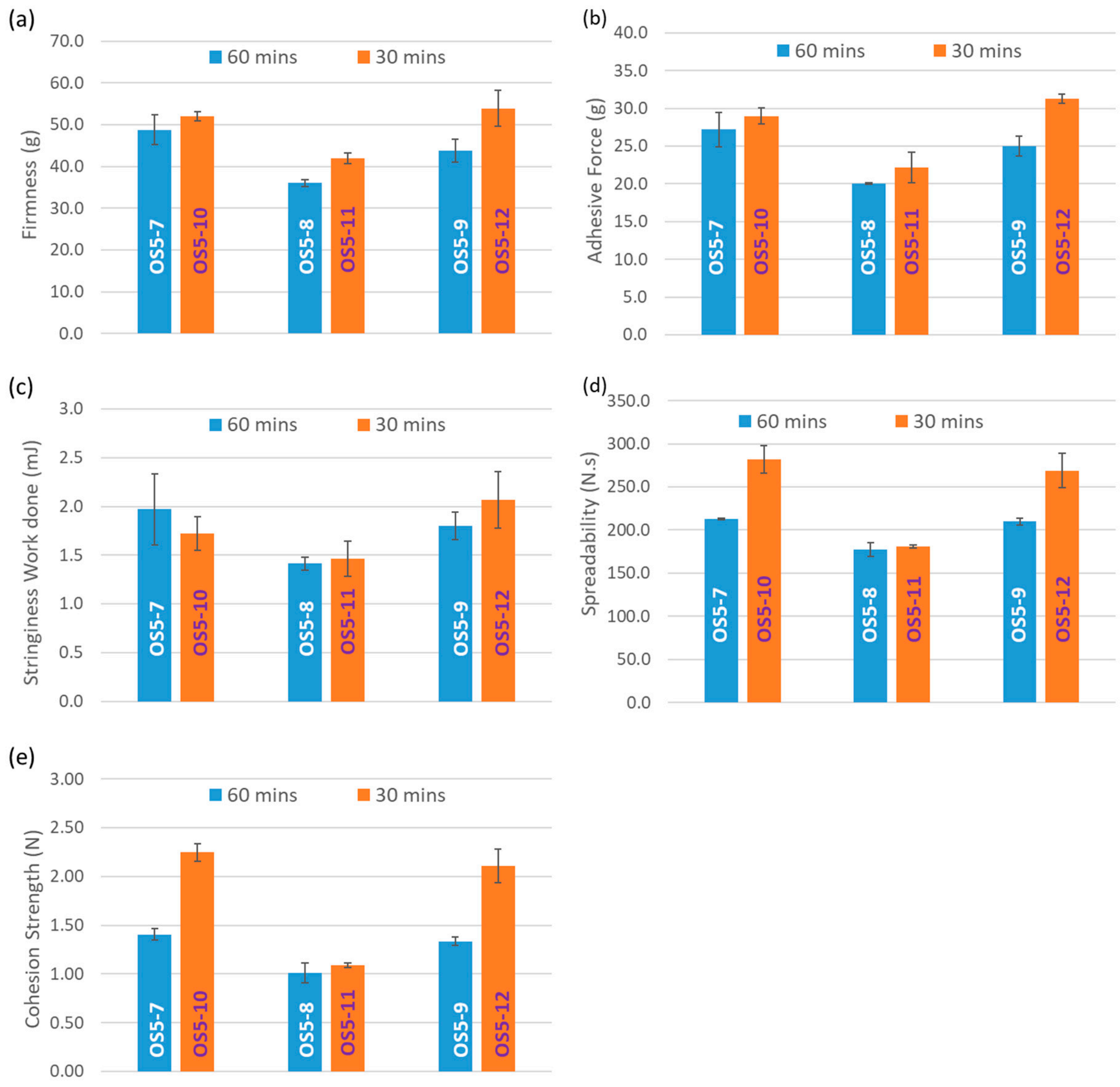

Figure S4. (a–e) Effect of PG addition mixing time at 46°C on textural properties of the ointments.

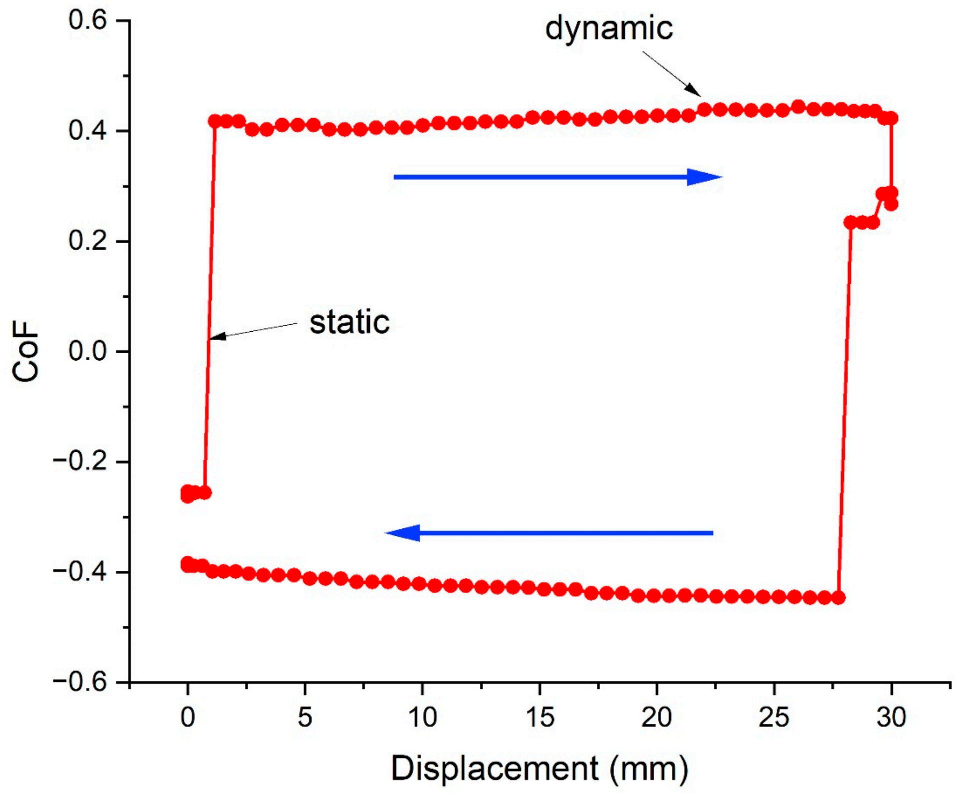

Figure S5. Coefficient of friction vs. probe displacement obtained during tribological experiment on a bioskin plate without ointment.

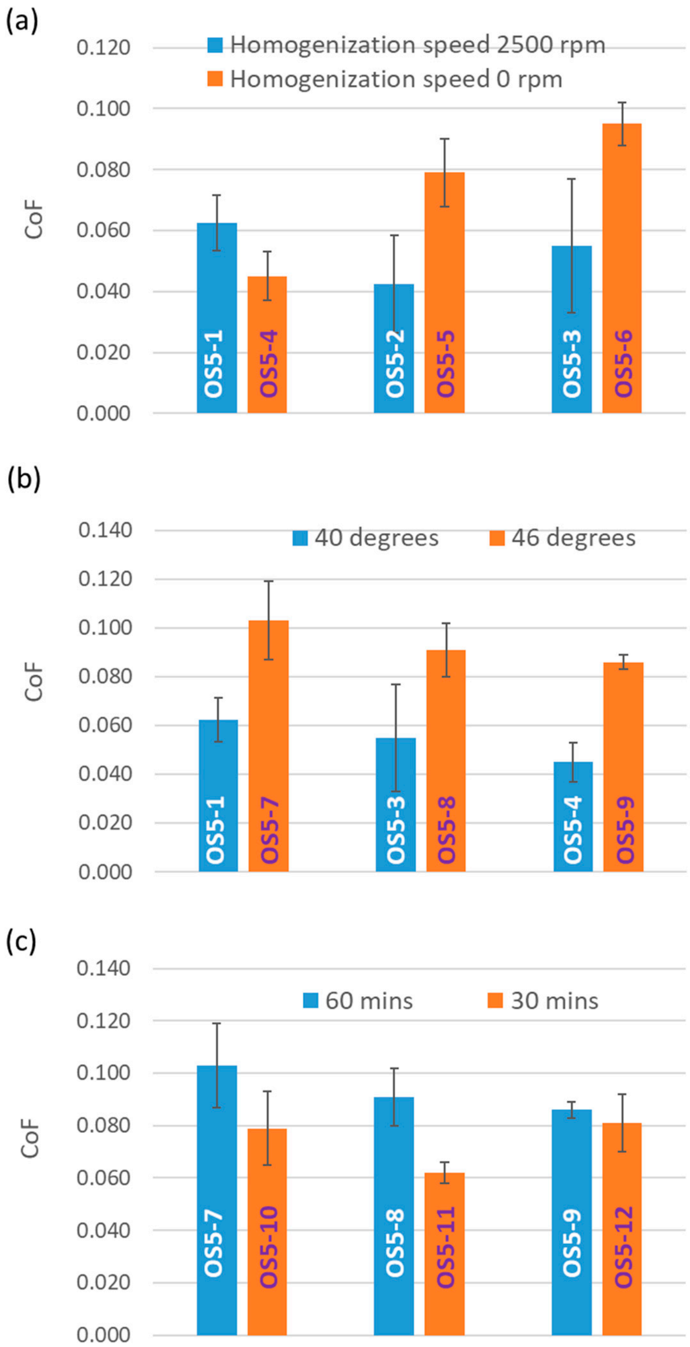

Figure S6. Variation of coefficients of friction with (a) step 5 homogenisation speed, (b) PG addition temperature, (c) PG addition mixing time.
